# Supplementary material for: From sequence to enzyme mechanism using multi-label machine learning
Source: BMC Bioinformatics. 2014 May 19;15:150. doi: 10.1186/1471-2105-15-150 (PMC4229970; doi:10.1186/1471-2105-15-150)
Supplement: Additional file 2 — Java code of ml2db. Additional file ml2db_code.tar.gz contains the Java source code to run the multi-label machine learning experiments and save the results to database. The code’s Javadoc is included. [file 1471-2105-15-150-S2.zip › additional file 2/ml2db/ml2db/doc/mulan/classifier/MultiLabelLearnerBase.html]

MultiLabelLearnerBase


JavaScript is disabled on your browser.


- Overview
- Package
- Class
- Use
- Tree
- Deprecated
- Index
- Help

- Prev Class
- Next Class

- Frames
- No Frames

- All Classes

- Summary:
- Nested |
- Field |
- Constr |
- Method

- Detail:
- Field |
- Constr |
- Method


mulan.classifier

## Class MultiLabelLearnerBase

- java.lang.Object
- - mulan.classifier.MultiLabelLearnerBase

- All Implemented Interfaces:
  :   java.io.Serializable, MultiLabelLearner, weka.core.TechnicalInformationHandler

  Direct Known Subclasses:
  :   BPMLL, MMPLearner, MultiLabelKNN, MultiLabelMetaLearner, TransformationBasedMultiLabelLearner

  ---

    

  ```
  public abstract class MultiLabelLearnerBase
  extends java.lang.Object
  implements weka.core.TechnicalInformationHandler, MultiLabelLearner, java.io.Serializable
  ```

  Common root base class for all multi-label learner types.
  Provides default implementation of `MultiLabelLearner` interface.

  Version:
  :   2010.12.27

  Author:
  :   Robert Friberg, Jozef Vilcek, Grigorios Tsoumakas

  See Also:
  :   Serialized Form

- - ### Constructor Summary

    Constructors

    Constructor and Description

    `MultiLabelLearnerBase()`
  - ### Method Summary

    Methods

    | Modifier and Type | Method and Description |
    | --- | --- |
    | `void` | `build(MultiLabelInstances trainingSet)` Builds the learner model from specified `MultiLabelInstances` data. |
    | `boolean` | `getDebug()` Get whether debugging is turned on. |
    | `abstract weka.core.TechnicalInformation` | `getTechnicalInformation()` Returns an instance of a TechnicalInformation object, containing detailed information about the technical background of this class, e.g., paper reference or book this class is based on. |
    | `abstract java.lang.String` | `globalInfo()` Returns a string describing the multi-label learner. |
    | `boolean` | `isUpdatable()` Returns value indicating if learner is updatable, so if learner is able to perform on-line learning. |
    | `MultiLabelLearner` | `makeCopy()` Creates a deep copy of the given learner using serialization. |
    | `MultiLabelOutput` | `makePrediction(weka.core.Instance instance)` Returns the prediction of the learner for a given input `Instance`. |
    | `void` | `setDebug(boolean debug)` Set debugging mode. |

    - ### Methods inherited from class java.lang.Object

      `equals, getClass, hashCode, notify, notifyAll, toString, wait, wait, wait`

- - ### Constructor Detail


    - #### MultiLabelLearnerBase

      ```
      public MultiLabelLearnerBase()
      ```
  - ### Method Detail


    - #### isUpdatable

      ```
      public boolean isUpdatable()
      ```

      **Description copied from interface: `MultiLabelLearner`**

      Returns value indicating if learner is updatable, so if learner is able to
      perform on-line learning. The fact if learner is updatable or not influences
      the behavior of `MultiLabelLearner.build(MultiLabelInstances)` method.  
      When `false` is returned, each call of the
      `MultiLabelLearner.build(MultiLabelInstances)` will initialize the learner from
      the scratch, removing any potential knowledge built by previously entered training data.
      When `true` is returned, then on the first call of the
      `MultiLabelLearner.build(MultiLabelInstances)` the learner is initialized
      with the passed training data. All other calls contribute to the existing learner's
      model with new data.

      **Specified by:**
      :   `isUpdatable` in interface `MultiLabelLearner`

      Returns:
      :   `true` if learner is updatable (on-line), `false` otherwise.


    - #### build

      ```
      public final void build(MultiLabelInstances trainingSet)
                       throws java.lang.Exception
      ```

      **Description copied from interface: `MultiLabelLearner`**

      Builds the learner model from specified `MultiLabelInstances` data.
      Sequential calls to this method either re-build the learners model with new data
      (off-line learner) or contribute to the existing model with new data (on-line learner).
      The behavior is determined by the outcome of `MultiLabelLearner.isUpdatable()` method.

      **Specified by:**
      :   `build` in interface `MultiLabelLearner`

      Parameters:
      :   `trainingSet` - set of training data, upon which the learner model should be built

      Throws:
      :   `java.lang.Exception` - if learner model was not created successfully
      :   `InvalidDataException` - if specified instances data is invalid or not supported by the learner

      See Also:
      :   `MultiLabelLearner.isUpdatable()`


    - #### makePrediction

      ```
      public final MultiLabelOutput makePrediction(weka.core.Instance instance)
                                            throws java.lang.Exception,
                                                   InvalidDataException,
                                                   ModelInitializationException
      ```

      **Description copied from interface: `MultiLabelLearner`**

      Returns the prediction of the learner for a given input `Instance`.

      **Specified by:**
      :   `makePrediction` in interface `MultiLabelLearner`

      Parameters:
      :   `instance` - the input given to the learner in the form of `Instance`

      Returns:
      :   a prediction of the learner in form of `MultiLabelOutput`.

      Throws:
      :   `java.lang.Exception` - if an error occurs while making the prediction.
      :   `InvalidDataException` - if specified instance data is invalid and can not be processed by the learner
      :   `ModelInitializationException` - if method is called before `MultiLabelLearner.build(MultiLabelInstances)`


    - #### setDebug

      ```
      public void setDebug(boolean debug)
      ```

      Set debugging mode.

      **Specified by:**
      :   `setDebug` in interface `MultiLabelLearner`

      Parameters:
      :   `debug` - `true` if debug output should be printed


    - #### getDebug

      ```
      public boolean getDebug()
      ```

      Get whether debugging is turned on.

      Returns:
      :   `true` if debugging output is on


    - #### makeCopy

      ```
      public MultiLabelLearner makeCopy()
                                 throws java.lang.Exception
      ```

      **Description copied from interface: `MultiLabelLearner`**

      Creates a deep copy of the given learner using serialization.

      **Specified by:**
      :   `makeCopy` in interface `MultiLabelLearner`

      Returns:
      :   a deep copy of the learner

      Throws:
      :   `java.lang.Exception` - if an error occurs while making copy of the learner.


    - #### getTechnicalInformation

      ```
      public abstract weka.core.TechnicalInformation getTechnicalInformation()
      ```

      Returns an instance of a TechnicalInformation object, containing detailed
      information about the technical background of this class, e.g., paper
      reference or book this class is based on.

      **Specified by:**
      :   `getTechnicalInformation` in interface `weka.core.TechnicalInformationHandler`

      Returns:
      :   the technical information about this class


    - #### globalInfo

      ```
      public abstract java.lang.String globalInfo()
      ```

      Returns a string describing the multi-label learner.

      Returns:
      :   a description suitable for displaying in a future gui


- Overview
- Package
- Class
- Use
- Tree
- Deprecated
- Index
- Help

- Prev Class
- Next Class

- Frames
- No Frames

- All Classes

- Summary:
- Nested |
- Field |
- Constr |
- Method

- Detail:
- Field |
- Constr |
- Method
